# Ganoderic Acid A Reverses Ultraviolet B Induced Hyperpigmentation via Multi-Targeted Regulation of Mitochondrial Homeostasis and Inflammation

**DOI:** 10.3390/molecules31152705

**Published:** 2026-08-04

**Authors:** Jingting Wang, Yuerong Qian, Qingna Gong, Rui He, Shanli Tian, Nannan Yu, Yanan Xi, Qiqi Wu, Guang-Li Wang, Jing Wang

**Affiliations:** 1 School of Chemical and Material Engineering, School of Cosmetic Science, Jiangnan University, Wuxi 214122, China; 7240610005@stu.jiangnan.edu.cn (J.W.); yuerongqian@outlook.com (Y.Q.); 7230610003@stu.jiangnan.edu.cn (Q.G.); 6240610019@stu.jiangnan.edu.cn (N.Y.); xiyanantt@163.com (Y.X.); wuqiqiqq@163.com (Q.W.); 2 Beijing Dr Plant Biotechnology Co., Ltd., Beijing 100032, China; herui@drplant.com (R.H.); tiansl@drplant.com.cn (S.T.)

**Keywords:** ultraviolet B, ganoderic acid A, melanogenesis, mitochondrial homeostasis, tyrosinase

## Abstract

Background: Conventional tyrosinase (TYR) inhibitors irritate skin and trigger rebound pigmentation, necessitating safer and more effective depigmenting agents. Methods: Biocompatibility was assessed by cell viability. Melanin content and TYR activity were measured spectrophotometrically. Reactive oxygen species (ROS), adenosine triphosphate (ATP), and inflammatory cytokines were detected by fluorescence, luminescence, and ELISA. Western blot and RT-qPCR assessed oxidative stress, inflammatory, and melanogenic targets. Molecular docking simulated Ganoderic Acid A (GAA) interactions with key proteins. Results: GAA exhibits good biocompatibility, inhibits melanin synthesis and TYR activity in B16-F10 cells, and reverses ultraviolet B-induced pigmentation. Mechanistically, GAA restores mitochondrial homeostasis by scavenging ROS, replenishing ATP, activating the nuclear factor erythroid 2-related factor 2 (Nrf2) axis, and inhibiting nuclear factor kappa-B (NF-κB) and cytokines such as tumor necrosis factor-α (TNF-α) and interleukin-6 (IL-6) to regulate the inflammatory microenvironment. This synergistic regulation inhibits the mitogen-activated protein kinase (MAPK) signaling pathway and down-regulates the microphthalmia-associated transcription factor (MITF) transcriptional network and the expression of TYR, tyrosinase-related protein 1 (TRP-1), and tyrosinase-related protein 2 (TRP-2). Conclusion: GAA eliminates ultraviolet B-induced hyperpigmentation through a multi-target mechanism of mitochondrial repair, inflammation inhibition, and direct binding to tyrosinase, and is a potential natural candidate drug for the treatment of skin diseases.

## 1. Introduction

Abnormal skin pigmentation, which is a common skin problem driven by various factors such as ultraviolet (UV) radiation, inflammation, and aging, is often clinically manifested as uneven skin color and refractory stains [1,2]. As the main environmental stressor, excessive ultraviolet B (UVB, 280–315 nm) exposure can strongly activate the microphthalmia-associated transcription factor (MITF) and related signaling pathways in melanocytes [2,3]. This intense stimulation drives excessive melanin synthesis and transport, ultimately resulting in adverse consequences including uneven skin tone, persistent dark spots, and premature photoaging [4]. To address these issues and restore skin homeostasis, effective skin-lightening products have become essential in cosmetic and dermatological care. Current conventional whitening schemes frequently utilize active agents such as kojic acid (KA) [5], arbutin [6], and resorcinol [7]. However, their efficacy primarily relies on the single-target mechanism of directly inhibiting tyrosinase (TYR). This singular pathway presents notable limitations, including an inability to fundamentally repair the oxidative stress-disrupted cellular microenvironment, susceptibility to skin irritation, and a high risk of rebound pigmentation following discontinuation [7,8]. Therefore, there is an urgent need to develop novel, safe and multi-target whitening agents to overcome the inherent defects of traditional therapies.

Excessive skin exposure to UVB radiation induces a massive accumulation of reactive oxygen species (ROS), causing severe oxidative stress, which in turn destroys cell homeostasis from the root cause, resulting in mitochondrial dysfunction and local inflammation [9]. Crucially, both mitochondrial impairment and the resulting inflammatory microenvironment are intimately linked to abnormal melanogenesis [10,11,12]. In this process, UVB-induced mitochondrial damage is an important upstream event, characterized by excessive production of mitochondrial ROS (mtROS) and adenosine triphosphate (ATP) depletion [13,14], which in turn activates inflammatory pathways such as nuclear factor kappa-B (NF-κB) [15,16,17], promotes the mitogen-activated protein kinase (MAPK)/microphthalmia-associated transcription factor (MITF) signaling axis, and accelerates melanin synthesis [18,19]. At present, most of the commercially available depigmentation agents focus on individual isolated downstream targets in the melanin production cascade [5,6,20]. Research emphasizing interventions at the source, namely restoring ROS-disrupted skin homeostasis to fundamentally inhibit melanogenesis, remains relatively scarce. Therefore, if mitochondrial homeostasis can be restored by activating the endogenous nuclear factor erythroid 2-related factor 2 (Nrf2) pathway, thereby synergistically regulating the intertwined pigmentation network, this multi-target intervention strategy will provide new ideas for inhibiting abnormal pigmentation.

*Ganoderma lucidum* has been a highly respected edible and medicinal fungus since ancient times. Modern research has extended its application to functional foods, dietary supplements and skin care. Its rich triterpenoids are regarded as an important material basis for pleiotropic biological activity. Ganoderic Acid A (GAA) is a representative triterpenoid active ingredient extracted from it [21,22,23], which has both antioxidant and cytoprotective properties, good biosafety and low skin irritation [22]. Compared with commonly studied pentacyclic triterpenoids such as asiatic acid [24] or ursolic acid [25], GAA features a distinct tetracyclic skeleton with specific hydroxyl and carboxyl substituents [26,27]. These structural characteristics may enhance its lipophilicity and electron-transfer capacity, potentially leading to favorable bioavailability and molecular interactions. These characteristics make GAA a natural candidate compound worthy of attention in the field of cosmetics. Current research underscores its potential to combat light-induced skin damage, mainly by protecting dermal fibroblasts and preventing collagen degradation [28,29,30]. However, despite accumulating evidence of these anti-aging aspects, the specific regulation of GAA on epidermal melanocytes and melanogenesis cascades is still uncharacterized [29]. Whether GAA can transform its own antioxidant advantages into a multi-target intervention strategy to reverse UVB-induced pigmentation by synergistically regulating mitochondrial homeostasis and inflammatory microenvironment is still a question worthy of further exploration.

In this study, we employed a UVB-irradiated B16-F10 melanocyte model combined with molecular docking simulation to systematically explore the mechanisms underlying the protective effects of GAA against UVB-triggered pigmentation. The working hypothesis is that the depigmenting action of GAA is not solely dependent on direct tyrosinase inhibition, but rather involves the restoration of mitochondrial homeostasis and the suppression of inflammatory cascades to repair the damaged cellular microenvironment through multi-target pathways. By investigating these potential synergistic regulatory networks, this study aims to lay a solid scientific foundation for the development of GAA as an advanced multifunctional active ingredient in dermatological treatments and skin-whitening cosmetic formulations.

## 2. Skin-Whitening Results

### 2.1. Evaluation of Cell Safety and Inhibitory Effects of GAA on Melanogenesis

**Figure 1:**
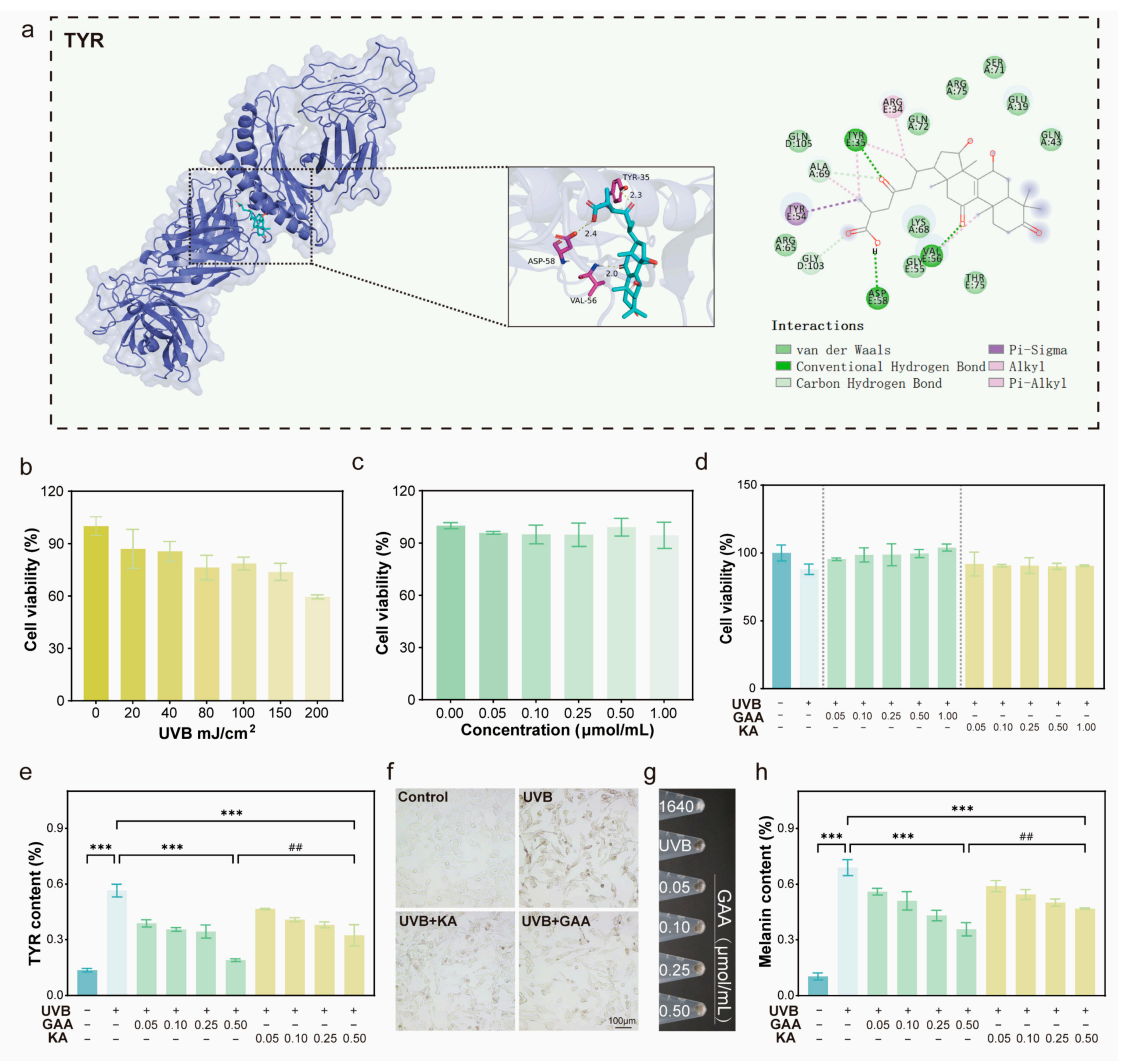
Molecular docking and anti-melanogenic effects of GAA. (**a**) Molecular docking model and 2D interaction diagram of GAA within the binding pocket of tyrosinase. Key residues (TYR-35, ASP-58, VAL-56) and interaction types (H-bonds, van der Waals, etc.) are indicated. In the enlarged view, the blue ball-and-stick model represents GAA; the purple one highlights key amino acid residues. (**b**) Cytotoxicity assay of UVB radiation (0–200 mJ/cm^2^) on cell viability measured by CCK-8 assay. (**c**) Cytotoxicity assay of GAA (0–1.00 μmol/mL) on cell viability measured by CCK-8 assay. (**d**) Effects of KA and GAA on the viability of UVB-irradiated cells. (**e**) Quantitative analysis of TYR content. (**f**) Representative cell morphology images (Scale bar = 100 μm). (**g**) Visual observation of cell pellets showing melanin pigmentation across different groups. (**h**) Total melanin content (%) quantification. Data are presented as mean ± SD (*n* = 3). *** *p* < 0.001 (vs. UVB-irradiated group). ## *p* < 0.01 (vs. UVB + KA group).

Molecular docking simulation provides a theoretical basis for the structural interaction between GAA and TYR. The results showed that GAA was accurately docked to the catalytic pocket of TYR protein, and the binding affinity was −8.9 kcal/mol. The stable conformation is mediated by hydrogen bonds and hydrophobic interactions, involving amino acid residues such as Tyr-35, Asp-58 and Val-56 (Figure 1a).

In order to evaluate the physiological efficacy, the conditions of UVB-induced pigmentation model were optimized [31]. B16-F10 cells were exposed to different intensities of UVB (0–200 mJ/cm^2^), and the dose of 80–100 mJ/cm^2^ was selected as the best range, because it can successfully induce melanin production stress, while maintaining cell viability at about 75–80%, avoiding excessive phototoxic cell death (Figure 1b). Biocompatibility evaluation confirmed that GAA had no cytotoxicity in the concentration range of 0.05–1.00 μmol/mL and had high safety (Figure 1c). In the UVB damage model, GAA was superior to the positive control KA in reducing radiation-induced cytotoxicity and restoring cell viability (Figure 1d).

In addition, GAA showed a strong dose-dependent inhibitory effect on melanin production. Intracellular quantitative detection showed that TYR activity was significantly decreased after GAA treatment (Figure 1e). Microscopic observation also confirmed the above results. UVB irradiation caused severe morphological stress and visible melanin accumulation, while GAA-treated cells maintained a healthy adherent state and significantly reduced intracellular pigmentation (Figure 1f). This anti-melanogenesis activity was also obvious in the direct observation of cell precipitation. With the increase in GAA concentration, the cell mass changed from dense black to light gray (Figure 1g). Quantitative analysis of total melanin content confirmed a robust dose-dependent downward trend, and the inhibitory effect of GAA was comparable to that of KA (Figure 1h).

### 2.2. Mitigation of UVB-Induced Oxidative Stress and Mitochondrial Dysfunction by GAA

**Figure 2:**
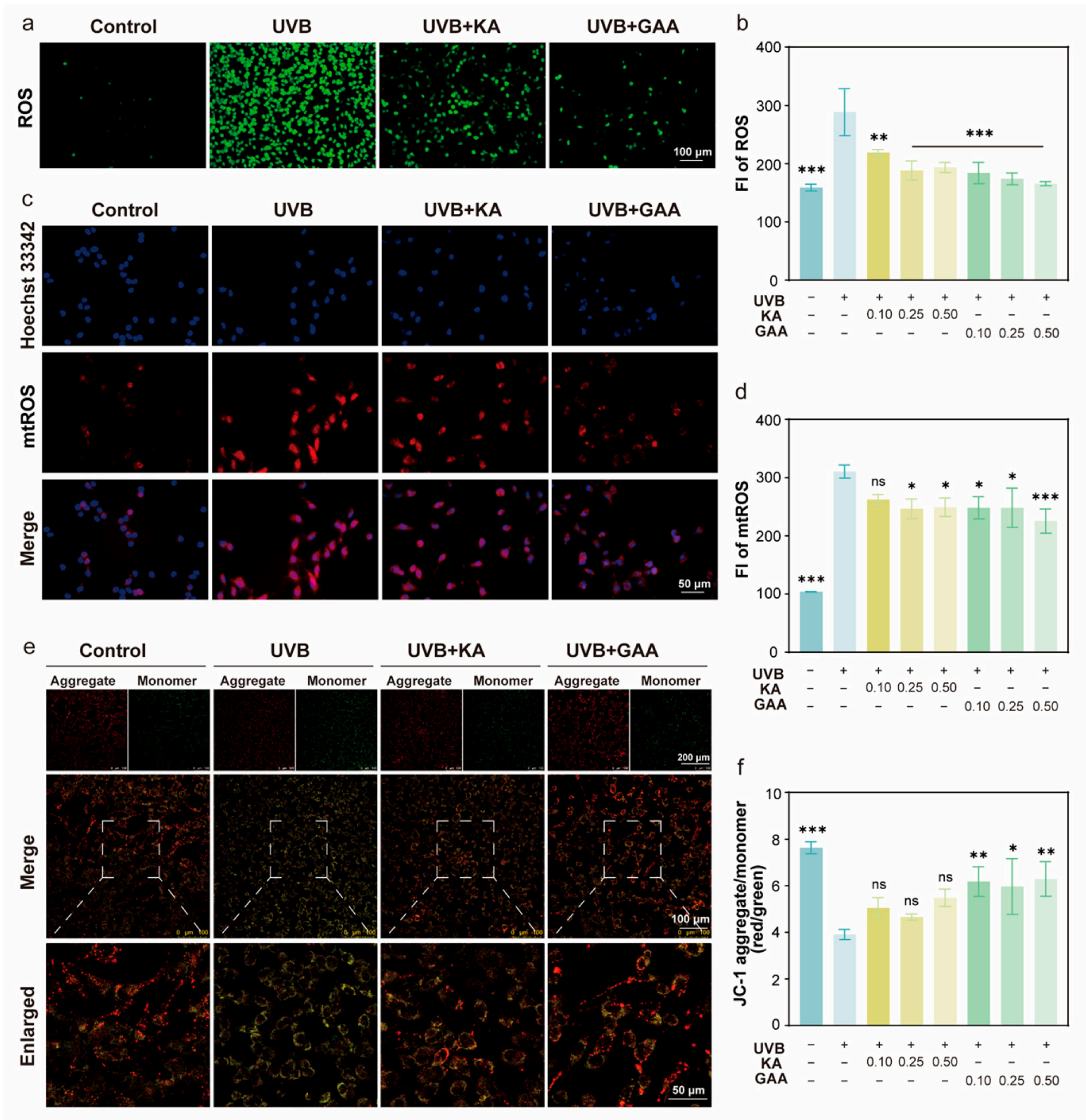
Regulatory effects of GAA on UVB-induced oxidative stress and mitochondrial membrane potential in B16-F10 cells. (**a**) Fluorescence imaging of total intracellular ROS levels treated with 0.5 μmol/mL KA or GAA (scale bar = 100 μm). (**b**) Effect of varying KA and GAA concentrations (0.1–0.5 μmol/mL) on relative total ROS fluorescence intensity (*n* = 3). (**c**) Fluorescence imaging of mtROS levels (scale bar = 50 μm). (**d**) Regulation of relative mtROS fluorescence intensity (*n* = 3). (**e**) JC-1 staining for MMP (JC-1 aggregates in red and JC-1 monomers in green, scale bar = 50–200 μm). Enlarged views of the areas within the white dashed boxes are shown in the third row. (**f**) Impact on JC-1 red/green fluorescence ratio (*n* = 3). Data are presented as mean ± SD (*n* = 3). * *p* < 0.05, ** *p* < 0.01, *** *p* < 0.001 (vs. UVB-irradiated group); ns indicates no significant difference (*p* > 0.05).

UVB irradiation can induce severe oxidative stress in B16-F10 cells [32,33], which is manifested by the accumulation of total intracellular ROS and the increase in mtROS (Figure 2a,c). Microplate fluorescence quantitative analysis showed that after treatment with GAA (0.10–0.50 μmol/mL), both oxidation markers decreased in a dose-dependent manner. Although both KA and GAA could effectively scavenge total ROS (Figure 2b; Supplementary Figure S1), the ability of GAA to scavenge mitochondrial local mtROS was significantly better than that of KA, especially at high doses of 0.50 μmol/mL (*** *p* < 0.001) (Figure 2d; Supplementary Figure S2).

Mitochondrial membrane potential (MMP) was detected by JC-1 staining to further evaluate mitochondrial structural integrity [34,35]. UVB irradiation caused a severe collapse of MMP, manifested as a significant transition from red (aggregates) to green (monomers) fluorescence (Figure 2e). Quantitative analysis of the red/green ratio confirmed that the ratio of the UVB model group decreased sharply. Within the measured dose range, KA treatment failed to make a statistically significant recovery of MMP, while GAA treatment effectively and dose-dependently reversed this depolarization, which was significantly better than KA (Figure 2f; Supplementary Figure S3).

Severe mitochondrial depolarization inevitably compromises cellular energy metabolism [34]. UVB radiation induced significant intracellular ATP depletion (Supplementary Figure S4) and abnormal stress-induced extracellular ATP leakage (Supplementary Figure S5). Unlike KA, GAA (0.50 μmol/mL) successfully restored intracellular ATP levels (** *p* < 0.01). Furthermore, GAA inhibited extracellular ATP leakage in a dose-dependent manner, and its inhibitory effect was significantly stronger than that of KA at the same concentration (# *p* < 0.05).

### 2.3. Enhancement of Antioxidant Defense via Nrf2 Pathway Activation by GAA

**Figure 3:**
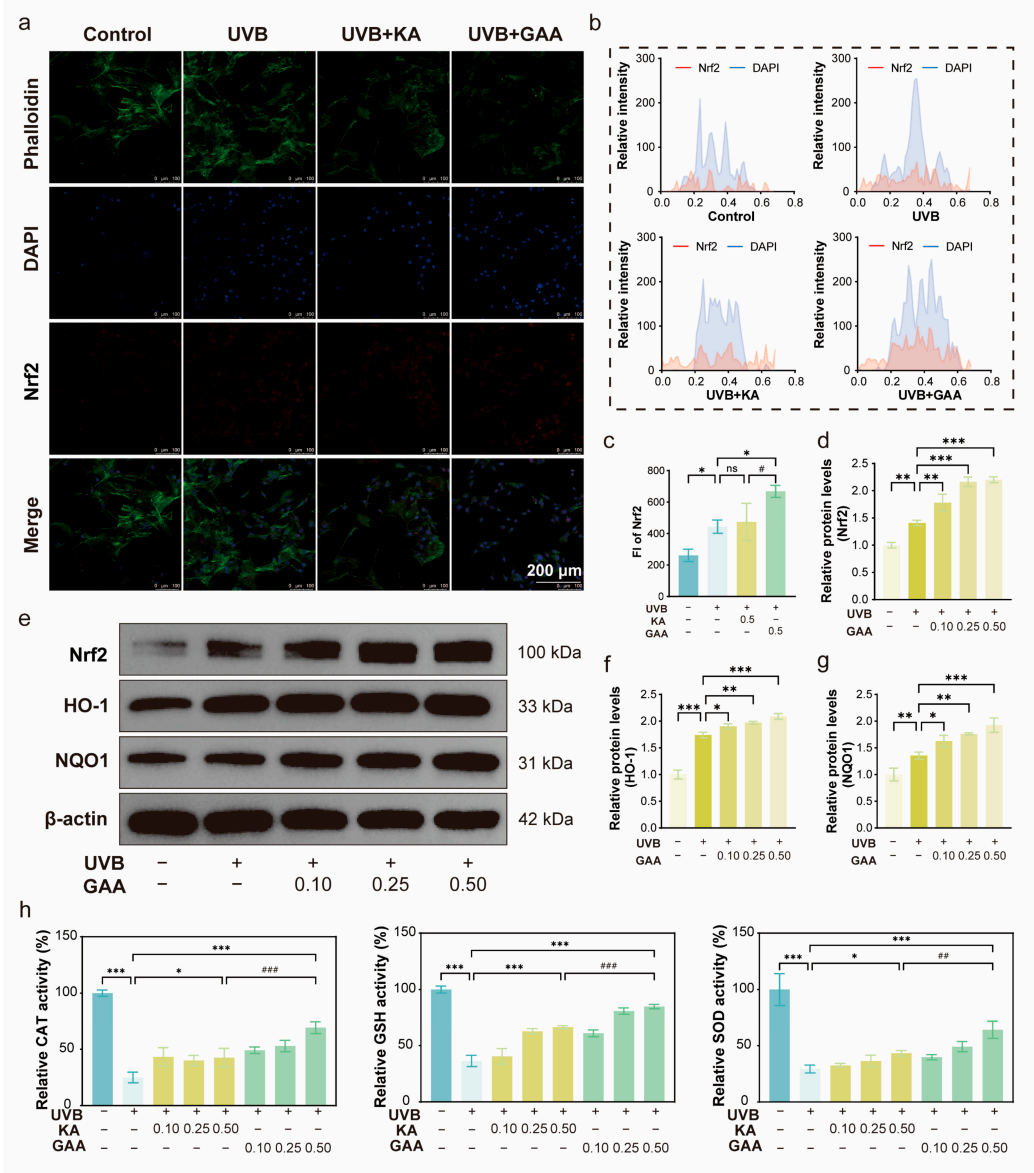
Mechanistic analysis of GAA enhancing antioxidant capacity via Nrf2 pathway activation. (**a**) Immunofluorescence observation of Nrf2 expression and nuclear translocation (scale bar = 200 μm). (**b**) Spatial fluorescence intensity profiling demonstrating the colocalization of Nrf2 (red) and DAPI (blue). (**c**) Quantitative analysis of Nrf2 fluorescence intensity (*n* = 3). (**d**) Quantitative analysis of Nrf2 protein expression levels (*n* = 3). (**e**) Western blot bands for Nrf2, HO-1, and NQO1. (**f**) Quantitative analysis of HO-1 protein expression levels (*n* = 3). (**g**) Quantitative analysis of NQO1 protein expression levels (*n* = 3). (**h**) Effect of KA and GAA (0.1–0.5 μmol/mL) on relative CAT activity, GSH content, and total SOD activity (*n* = 3). Data are presented as mean ± SD (*n* = 3). * *p* < 0.05, ** *p* < 0.01, *** *p* < 0.001 (vs. UVB-irradiated group); # *p* < 0.05, ## *p* < 0.01, ### *p* < 0.001 (vs. UVB + KA group). ns indicates no significant difference (*p* > 0.05).

To elucidate the molecular mechanism of GAA antioxidant effect, the regulation of Nrf2 signaling pathway was investigated. Immunofluorescence staining (Figure 3a) and fluorescence intensity quantification (Figure 3c) showed that GAA treatment (0.50 μmol/mL) enhanced Nrf2 protein accumulation, which was statistically superior to KA. Spatial fluorescence analysis (Figure 3b) demonstrated a high colocalization of the Nrf2 signal (red) with the nuclear stain DAPI (blue) in the GAA-treated group, confirming that GAA could strongly induce Nrf2 nuclear translocation. After translocation, activated Nrf2 drives the transcription of protective phase II enzymes. Western blot analysis further confirmed the above fluorescence results, indicating that GAA up-regulated the total Nrf2 protein expression in a dose-dependent manner (Figure 3d–g). Consequently, compared with the UVB model group, GAA significantly and dose-dependently increased the protein levels of the key downstream target molecules HO-1 and NQO1.

The functional effect of Nrf2 pathway activation was verified by detecting endogenous antioxidant enzyme activity and lipid peroxidation level [36]. UVB irradiation severely inhibited the activity of intracellular catalase (CAT), glutathione (GSH), total superoxide dismutase (SOD), and induced the excessive production of malondialdehyde (MDA), the main biomarker of lipid peroxidation [37]. GAA treatment dose-dependently reversed these changes and effectively reduced MDA levels (Supplementary Figure S6). At the highest concentration (0.50 μmol/mL), the ability of GAA to restore CAT, GSH and total SOD activity was significantly better than that of KA (Figure 3h).

### 2.4. Regulation of UVB-Induced Inflammation and MAPK Pathways by GAA

**Figure 4:**
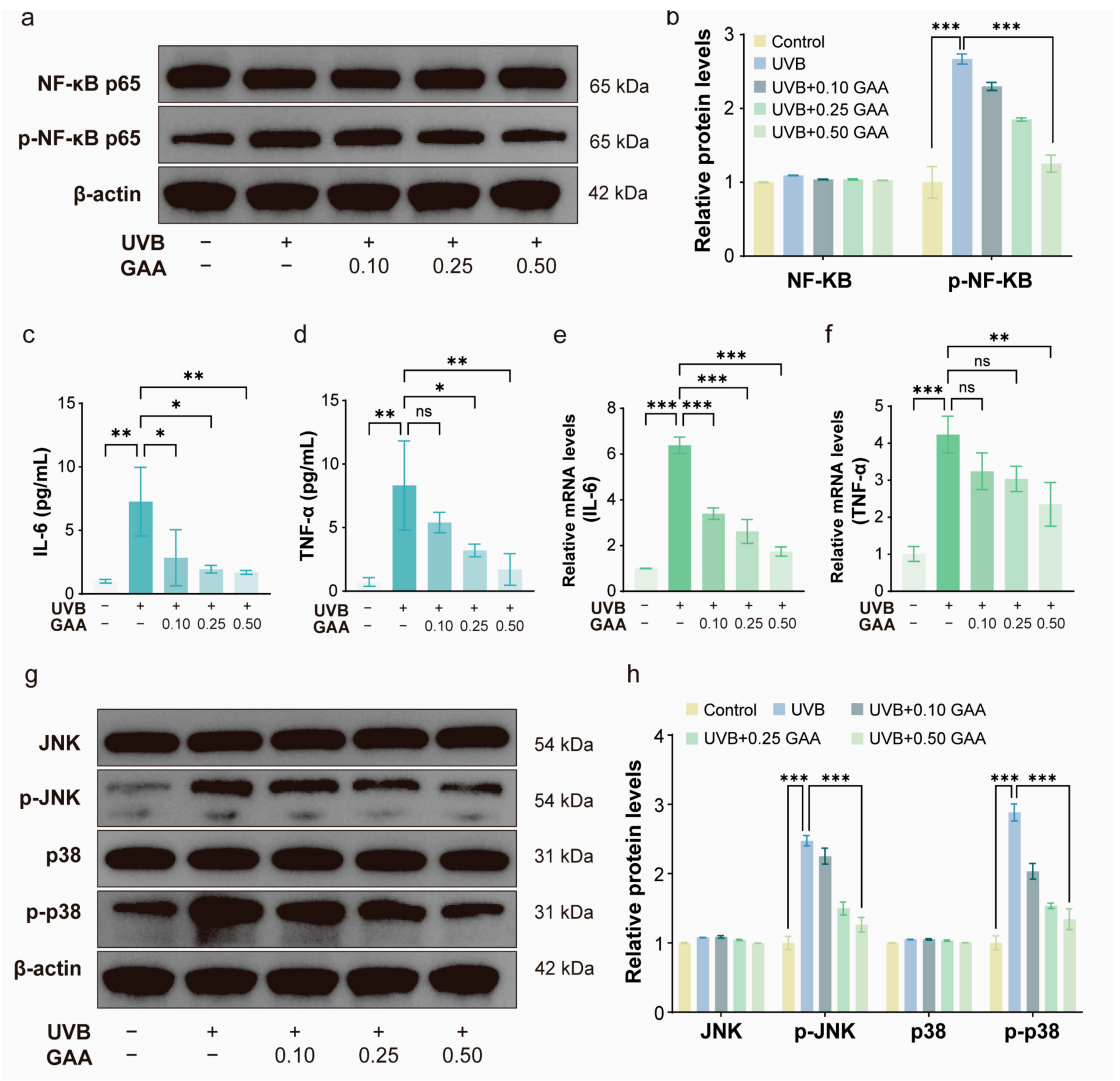
Inhibitory effects of GAA on NF-κB activation, inflammatory cytokine expression, and MAPK phosphorylation induced by UVB. (**a**) Western blot bands for NF-κB p65 and p-NF-κB p65. (**b**) Quantitative analysis of relative NF-κB p65 and p-NF-κB p65 expression (*n* = 3). (**c**) ELISA detection of IL-6 protein release (pg/mL, *n* = 3). (**d**) ELISA detection of TNF-α protein release (pg/mL, *n* = 3). (**e**) Impact of GAA on *IL-6* mRNA levels (*n* = 3). (**f**) Impact of GAA on *TNF-α* mRNA levels (*n* = 3). (**g**) Western blot bands for JNK, p-JNK, p38, and p-p38. (**h**) Quantitative statistical analysis of JNK, p-JNK, p38, and p-p38 protein levels (*n* = 3). Data are presented as mean ± SD (*n* = 3). * *p* < 0.05, ** *p* < 0.01, *** *p* < 0.001; ns indicates no significant difference (*p* > 0.05).

Oxidative stress induced by UVB is closely related to inflammatory response [38,39,40]. Western blot results showed that GAA intervention could effectively block UVB-induced NF-κB p65 phosphorylation (Figure 4a,b). Correspondingly, GAA significantly and dose-dependently suppressed both the secretion (Figure 4c,d) and mRNA expression (Figure 4e,f) of the pro-inflammatory cytokines tumor necrosis factor-α (TNF-α) and interleukin-6 (IL-6). By alleviating this inflammatory cascade, GAA weakens its microenvironmental factors that promote melanin production. In addition, GAA also plays a positive regulatory role in MAPK signaling pathway. UVB irradiation significantly up-regulated the phosphorylation levels of c-Jun N-terminal kinase (JNK) and p38 mitogen-activated protein kinase (p38), and GAA treatment effectively reversed this effect (Figure 4g,h). The targeted downregulation of MAPK (JNK/p38) signaling contributes to the suppression of MITF, the master transcription factor for melanin synthesis [3,41].

### 2.5. Downregulation of MITF and Key Melanogenic Factors by GAA

**Figure 5:**
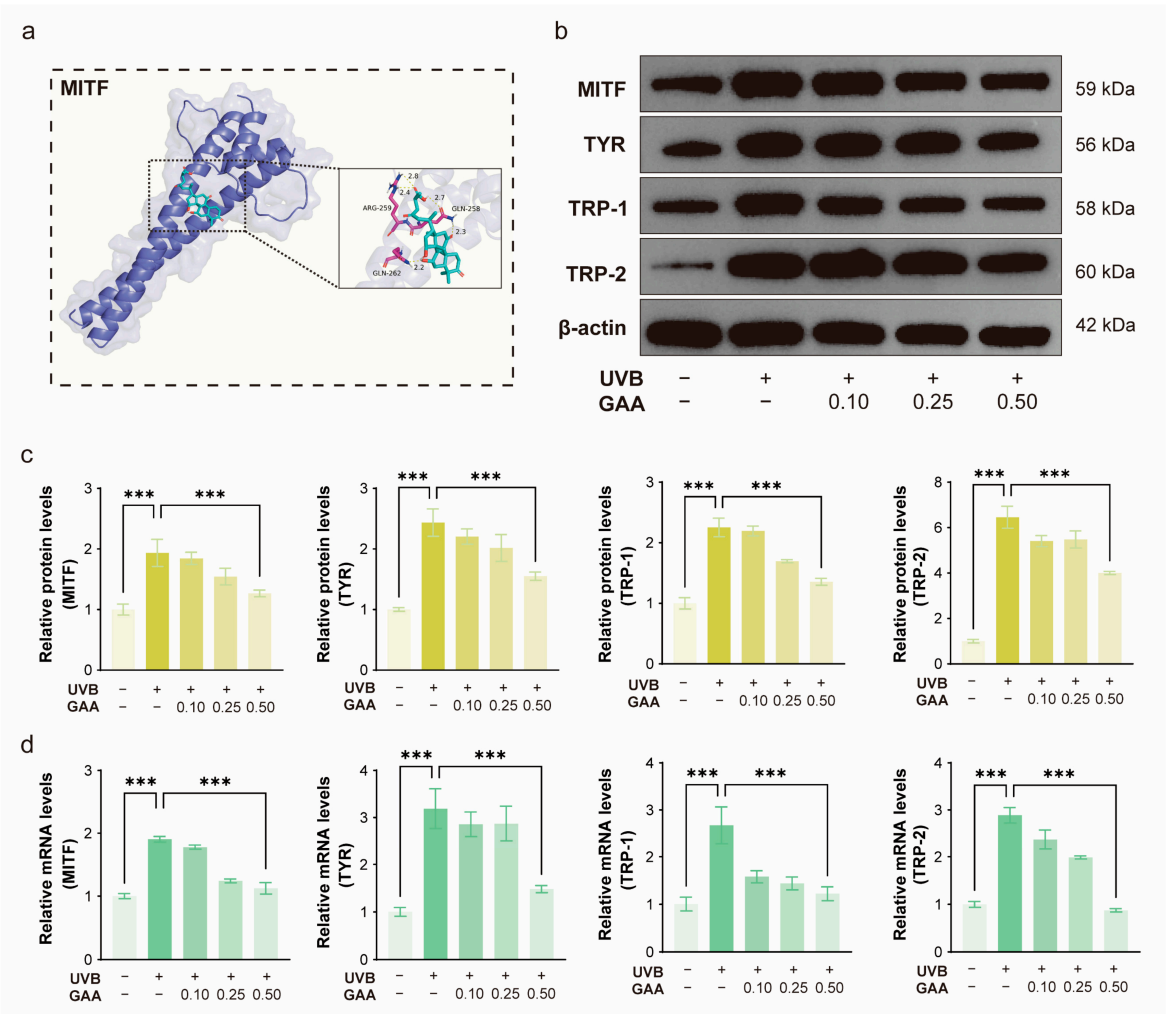
GAA regulates the expression of melanogenic genes and proteins in UVB-irradiated cells. (**a**) Molecular docking of GAA with MITF. Inset shows the detailed binding interactions with key amino acid residues (ARG-259, GLN-258, GLN-262), where the blue ball-and-stick model represents GAA and the purple one highlights these key residues (**b**) Representative Western blot images showing the protein levels of MITF, TYR, TRP1, and TRP2. (**c**) Densitometric quantification of the relative protein expression levels of MITF, TYR, TRP1, and TRP2 normalized to β-actin. (**d**) Quantitative RT-qPCR analysis of the mRNA expression levels of *MITF*, *TYR*, *TRP1*, and *TRP2*. Data are presented as mean ± SD (*n* = 3). *** *p* < 0.001.

By evaluating the regulation of GAA on MITF and its downstream signal cascades, the anti-melanogenesis mechanism was analyzed. Molecular docking (Figure 5a) showed that GAA formed stable hydrogen bonds through Arg-259, Gln-258 and Gln-262, and accurately bound to the MITF pocket (affinity of −6.9 kcal/mol), suggesting that GAA may directly regulate the MITF pathway. At the molecular level, Western blot analysis (Figure 5b,c) showed that UVB irradiation significantly up-regulated the protein expression of MITF and its downstream target enzymes (TYR, tyrosinase-related protein 1 (TRP-1), and tyrosinase-related protein 2 (TRP-2)), while GAA treatment could reverse this overexpression in a dose-dependent manner. RT-qPCR analysis (Figure 5d) confirmed that GAA could effectively inhibit the transcriptional activation of *MITF*, *TYR*, *TRP-1* and *TRP-2* mRNA induced by UVB.

## 3. Discussion

This study investigated the molecular mechanism of GAA anti-melanogenesis in UVB-induced B16-F10 cell model. It was found that GAA had a synergistic blocking effect on oxidative stress, inflammatory response and melanogenesis-related signaling pathways by inhibiting intracellular melanin accumulation and TYR activity, confirming that GAA can directly interfere with the initial step of melanin biosynthesis by targeting tyrosinase activity. These findings suggest that GAA has the potential to be a safe and efficient multi-target photo-induced pigmentation intervention candidate.

Beyond direct enzyme inhibition, restoring cellular redox balance is also critical for long-term reversal of pigmentation. The results showed that UVB radiation could destroy mitochondrial homeostasis, resulting in local increase in mtROS, MMP collapse and ATP depletion [42]. GAA treatment actively scavenged mtROS, stabilized MMP, and restored ATP levels, highlighting its pronounced mitochondrial-targeting and protective capacity. The interplay between mitochondrial dysfunction and oxidative stress is not a simple primary-versus-secondary relationship, but rather constitutes a self-amplifying positive-feedback loop: UVB-induced global oxidative stress damages mitochondria, and the resulting mitochondrial damage in turn triggers excessive mtROS release, thereby perpetuating a vicious cycle [43,44]. Within this framework, the ability of GAA to scavenge mtROS and stabilize MMP represents a direct intervention at the core amplification node of this stress network, with significantly greater efficacy than that of KA. Mechanistically, this mitochondrial protection is primarily achieved through the promotion of Nrf2 nuclear translocation. As a master transcriptional regulator of cellular antioxidant responses, Nrf2 drives the expression of downstream phase II detoxifying enzymes. In this study, GAA dose-dependently induced the expression of HO-1 and NQO1, which together suppress superoxide anion generation and facilitate the detoxification of oxidative intermediates [16,19]. This induction of key antioxidant enzymes effectively restored the endogenous defense network, as evidenced by increased activities of SOD, CAT, and GSH, and decreased MDA accumulation [45,46,47]. Collectively, these findings confirm that GAA attenuates UVB-induced oxidative damage by actively promoting Nrf2 nuclear translocation and driving a broad-spectrum antioxidant defense system. The restoration of mitochondrial integrity is not merely a passive response to oxidative stress, but rather a structural hub that breaks the vicious cycle of mitochondrial damage, energy crisis, and inflammatory amplification, thereby effectively intercepting downstream melanogenic signaling. This targeted ROS scavenging strategy not only rescues mitochondrial energy metabolism but also lays the biochemical foundation for blocking subsequent melanin synthesis.

Beyond its antioxidant action, GAA also coordinately regulates the inflammatory and melanogenic signaling cascades, thereby restoring the broader cellular microenvironment disrupted by UVB. Our findings illustrate that GAA optimizes the local microenvironment by neutralizing UVB-induced inflammatory cascades. GAA treatment inhibited the phosphorylation of NF-κB p65, thereby preventing the secretion and transcriptional activation of pro-inflammatory cytokines, specifically IL-6 and TNF-α [48,49]. As paracrine signaling molecules, these cytokines further promote overactivation of melanocytes, highlighting the critical importance of suppressing the inflammatory microenvironment. Concurrently, GAA can inhibit the stress-activated MAPK (JNK/p38) pathway. By disrupting this stress-nuclear signaling loop, GAA synergistically down-regulated the transcription and translation levels of MITF, a key factor regulating melanocyte development [50,51,52]. Molecular docking simulation also revealed that GAA can directly form stable hydrogen bonds in the MITF binding pocket, pointing to a dual anti-melanogenic mechanism whereby GAA not only suppresses upstream stress signaling but also directly interferes with the MITF protein structure, thereby cooperatively inhibiting the entire MITF/TYR/TRP transcription and translation axis. With the down-regulation of MITF, the expression of TYR, TRP-1 and TRP-2 was inhibited, which are necessary for stabilizing the melanosome complex and determining melanin composition [7,8]. From a translational perspective, GAA holds distinct advantages over conventional depigmenting agents such as KA. Unlike single-target tyrosinase inhibitors, GAA combines low cytotoxicity with a multi-target regulatory capacity that simultaneously addresses oxidative stress, inflammation, and melanogenesis, offering a favorable balance between safety and efficacy.

Despite these promising in vitro findings, several limitations of the present study warrant acknowledgment. First, we did not employ gene knockdown or pharmacological inhibition approaches to directly establish the Nrf2 dependency of GAA’s protective effects. Second, ERK signaling, a MAPK family member with complex, context-dependent roles in melanogenesis [53,54], was not evaluated in this study. Incorporating ERK analysis in future work will help build a more complete picture of the MAPK regulatory network underlying GAA’s anti-melanogenic effects. Third, although B16-F10 cells serve as a well-established screening platform for melanogenesis research, their biological properties differ from those of human melanocytes, which limits the direct translational relevance of our findings to human skin physiology. Fourth, the effects of GAA on melanocyte dendricity and melanosome transfer remain unexplored; future studies employing human melanocyte–keratinocyte co-culture systems would be valuable to address these questions. Additionally, the lack of validation in in vivo UVB-induced pigmentation models represents another major limitation, and subsequent incorporation of such models is essential for translational development.

In summary, UVB-induced melanin production is often accompanied by severe oxidative stress, mitochondrial dysfunction and inflammatory response activation [37,55]. By activating the Nrf2 antioxidant axis and blocking the NF-κB and MAPK signaling branches, GAA effectively restores mitochondrial homeostasis, improves the inflammatory microenvironment, down-regulates MITF and tyrosinase protein levels, and exerts an efficient anti-melanogenesis effect. Regulation of this multi-target network enables concurrent tyrosinase inhibition and restoration of cellular homeostasis, representing a safe and promising new therapeutic approach for light-triggered pigmentary disorders.

## 4. Materials and Methods

### 4.1. Materials

Ganoderic acid A (GAA, purity ≥ 98%) was purchased from Energy Chemical (Shanghai, China). B16-F10 cells were obtained from the Peking Union Medical College Cell Resource Center (Beijing, China). DMEM, RPMI-1640, fetal bovine serum (FBS), EDTA, and PBS were sourced from Thermo Fisher Scientific (Waltham, MA, USA). Cell Counting Kit-8(CCK-8) was from Meilun Biology Technology (Guangzhou, China). Fluorescence probes (DCFH-DA, JC-1, MitoSOX Red) and ATP kits were from Beyotime Biotechnology (Shanghai, China). Assay kits for CAT, GSH, SOD and MDA, were from Nanjing Jiancheng Bioengineering Institute (Nanjing, China). TNF-α and IL-6 ELISAs were supplied by Elabscience (Wuhan, China). Primary antibodies used for Western blot analysis were as follows: Nrf2 (AF0639), HO-1 (AF5393), NQO1 (DF6437), NF-κB p65 (AF5006), p-NF-κB p65 (AF2006), JNK (AF6318), p-JNK (AF3318), p38 (AF6456), p-p38 (AF4001), TRP-2 (AF5303), and β-actin (AF7018) were purchased from Affinity Biosciences (Liyang, China); MITF (bs-1990R), TYR (bs-0819R), and TRP-1 (bs-15510R) were obtained from Bioss Antibodies (Beijing, China). All other chemicals utilized were of analytical grade.

### 4.2. Simulation of Direct Binding to Target Proteins

Molecular docking studies were performed to investigate the binding affinity and interaction modes of GAA with TYR (PDB: 7RK7) and MITF (PDB:7EOD). AutoDock Vina 1.2.6 is used for simulation calculation. The protein structure was pretreated with PyMOL 3.1 to remove water molecules and add polar hydrogen. The docking conformation with the lowest binding energy was selected to analyze the hydrogen bond and hydrophobic interaction modes.

### 4.3. Cell Viability and Establishment of the UVB-Induced Hyperpigmentation Model

B16-F10 cells were cultured in RPMI-1640 (or DMEM) containing 10% FBS and 1% penicillin-streptomycin at 37 °C and 5% CO_2_. To determine the optimal UVB irradiation dose and safe concentration range of GAA, CCK-8 assay was used to evaluate cell viability. For UVB dose optimization, B16-F10 cells were seeded in 96-well plates at a density of 1 × 10^4^ cells per well and cultured for 24 h to allow adherence. The cells were washed with PBS and irradiated with various doses of UVB (312 nm, 0–200 mJ/cm^2^) using a UV crosslinker (SCIENTZ03 PRO, Scientz Biotechnology, Ningbo, China). Immediately after irradiation, fresh culture medium was added, and the cells were incubated for an additional 24 h. For GAA cytotoxicity evaluation, B16-F10 cells were seeded in 96-well plates and treated with various concentrations of GAA (0.05–1.00 μmol/mL) for 24 h. Subsequently, CCK-8 reagent was added and incubated in the dark at 37 °C for 40 min. The absorbance was measured at 450 nm using a multifunctional microplate reader (Varioskan ALF, Thermo Fisher Scientific, Waltham, MA, USA). Cell viability was calculated relative to the untreated control group.

Based on the cell viability results, a UVB-induced hyperpigmentation reversal model was established. Briefly, B16-F10 cells in the logarithmic growth phase were seeded in 12-well plates at a density of 1.2 × 10^6^ cells/mL and cultured for 24 h to allow adherence. Medium was replaced with serum-free medium to prevent interference, and cells were washed with PBS and irradiated with 100 mJ/cm^2^ UVB. Then replaced with serum-containing medium with GAA (0.05–1.00 μmol/mL) or kojic acid (KA, positive control). Groups: Control, UVB model (UVB + vehicle), UVB + KA, UVB + GAA (*n* = 3 per group). Cells were incubated for 24 h before subsequent analyses.

### 4.4. Evaluation of Anti-Melanogenic Efficacy and Tyrosinase Activity

Melanin was extracted from cells by 1% Triton X-100. The cell precipitate was dissolved in 1.0 M NaOH solution containing 10% DMSO, reacted at 80 °C for 1 h, and the absorbance was recorded at 405 nm. In the detection of tyrosinase activity, the lysate supernatant was taken as the crude enzyme extract. 50 μL sample was mixed with 150 μL 10 mM L-DOPA, incubated at 37 °C for 30 min, and the enzyme activity was measured at 475 nm.

### 4.5. Assessment of Mitochondrial Homeostasis

The levels of total ROS and mtROS were detected by DCFH-DA and MitoSOX Red probes, respectively. MMP was assessed by JC-1 staining, and the red/green fluorescence ratio was quantified by laser scanning confocal microscopy (SP8, Leica Microsystems, Wetzlar, Germany). Fluorescence images were analyzed with ImageJ (ImageJ 1.54d). For each experimental condition, at least five fields were randomly selected from three independent experiments, and the average integrated optical density was normalized to the number of cells in each field. Intracellular ATP content was measured using a multifunctional microplate reader. All signals were normalized according to the sample protein concentration.

### 4.6. Assessment of Nrf2 Nuclear Translocation

To assess nuclear translocation of Nrf2, cells were fixed with 4% paraformaldehyde, permeabilized with 0.5% Triton X-100, and then incubated with Nrf2 primary antibody at 4 °C for 12 h. After washing with PBS, Cy3-labeled secondary antibody and FITC-phalloidin were added and treated in dark for 1 h. The nucleus was re-stained with DAPI for 15 min. Confocal microscopy was used to collect images, and band densitometry was performed using ImageJ software. All statistical analyses were performed based on quantitative data from three independent.

### 4.7. Evaluation of Oxidative Stress and Inflammatory Responses

The activity of total SOD, CAT and the levels of GSH and MDA in cell lysate were detected by commercial colorimetric kit. After centrifugation to remove the precipitate, the concentrations of IL-6 and TNF-α in the culture supernatant were measured by ELISA kit. The absorbance was recorded at 450 nm, and all biochemical indicators were normalized to protein concentration.

### 4.8. Evaluation of Melanogenic, Antioxidant, and Inflammatory Signaling Pathways

Cells were lysed with RIPA buffer containing 1% PMSF, and protein quantification was performed by BCA method. The same amount of protein (50 μg) was separated by 10% SDS-PAGE and transferred to PVDF membrane. The membrane was blocked with 5% skimmed milk powder for 1 h, and then incubated with primary antibodies of Nrf2, HO-1, NQO1, NF-κB p65, phospho-NF-κB p65, JNK, phospho-JNK, p38, phospho-p38, MITF, TYR, TRP-1, TRP-2 and β-actin at 4 °C overnight. After incubation with secondary antibody, the bands were collected by imaging system (ChemiDoc, Bio-Rad, Hercules, CA, USA). Band intensities were quantified using ImageJ. For each target protein, the integrated density of the band was normalized to that of β-actin in the same sample, and the relative protein levels was calculated as the fold change compared to the control group. Data from at least three independent experiments were averaged and expressed as mean ± SD before statistical analysis.

### 4.9. Analysis of Transcriptional Regulation in Melanogenesis and Inflammation

Total RNA was extracted by Trizol and reverse transcribed into cDNA. SYBR Green Master Mix was used to perform RT-qPCR on the real-time quantitative PCR system (CFX Duet, Bio-Rad, Hercules, CA, USA). The relative mRNA levels of *IL-6*, *TNF-α* and melanogenesis-related genes (*MITF*, *TYR*, *TRP-1*, *TRP-2*) were calculated by 2^−ΔΔCt^ method using GAPDH as an internal reference. The primer sequences used are shown in Table S1.

### 4.10. Statistical Analysis

All experiments were independently repeated three times, and the data are expressed as mean ± standard deviation (SD). Statistical analysis was performed using GraphPad Prism 9.5. Comparisons among multiple groups were conducted using one-way analysis of variance (ANOVA). In the figures, * *p* < 0.05, ** *p* < 0.01, and *** *p* < 0.001 indicate significant differences compared with the UVB-irradiated group; # *p* < 0.05, ## *p* < 0.01, and ### *p* < 0.001 indicate significant differences compared with the UVB + KA group; ns indicates no significant difference (*p* > 0.05). For fluorescence intensity quantification, at least five randomly selected fields per well were measured using ImageJ, and the average values were used for statistical analysis.

## Data Availability

The original contributions presented in this study are included in the article/Supplementary Materials. Further inquiries can be directed to the corresponding authors.

## Supplementary Materials

The following supporting information can be downloaded at https://www.mdpi.com/article/10.3390/molecules31152705/s1. Figure S1: Quantitative analysis of relative total ROS fluorescence intensity (*n* = 3); Figure S2: Quantitative analysis of relative mtROS fluorescence intensity (*n* = 3); Figure S3: Quantitative evaluation of the JC-1 aggregate/monomer fluorescence intensity ratio (*n* = 3); Figure S4: Intracellular ATP content (nmol/mg protein, *n* = 3); Figure S5: Extracellular ATP release (nmol/mg protein, *n* = 3); Figure S6: Effect of KA and GAA (0.1–0.5 μmol/mL) on relative MDA levels (nmol/mg protein, *n* = 3); Table S1: Primer sequences used for RT-qPCR analysis.

## Abbreviations

The following abbreviations are used in this manuscript:

ATP	adenosine triphosphate
CAT	catalase
CCK-8	Cell Counting Kit-8
ELISA	enzyme-linked immunosorbent assay
GAA	Ganoderic acid A
GSH	glutathione
HO-1	heme oxygenase-1
IL-6	interleukin-6
JC-1	5,5′,6,6′-tetrachloro-1,1′,3,3′-tetraethylbenzimidazolylcarbocyanine iodide
JNK	c-Jun N-terminal kinase
KA	kojic acid
MAPK	mitogen-activated protein kinase
MDA	malondialdehyde
MITF	microphthalmia-associated transcription factor
MMP	mitochondrial membrane potential
mtROS	mitochondrial reactive oxygen species
NF-κB	nuclear factor kappa-light-chain-enhancer of activated B cells
NQO1	NAD(P)H:quinone oxidoreductase 1
Nrf2	nuclear factor erythroid 2-related factor 2
p38	p38 mitogen-activated protein kinase
ROS	reactive oxygen species
RT-qPCR	reverse transcription quantitative polymerase chain reaction
SOD	superoxide dismutase
TNF-α	tumor necrosis factor-alpha
TRP-1	tyrosinase-related protein 1
TRP-2	tyrosinase-related protein 2
TYR	tyrosinase
UVB	ultraviolet B
