# Supplementary material for: Ganoderic Acid A Reverses Ultraviolet B Induced Hyperpigmentation via Multi-Targeted Regulation of Mitochondrial Homeostasis and Inflammation
Source: Molecules. 2026 Aug 4;31(15):2705. doi: 10.3390/molecules31152705 (PMC13467712; doi:10.3390/molecules31152705)
Supplement: Supplementary file 1 [file molecules-31-02705-s001.zip › molecules-4451230-supplementary.pdf]

Supplementary Materials

Table S1. Primer sequences used for RT-qPCR analysis.

Gene	Forward Primer (5' - 3')	Reverse Primer (5' - 3')
MITF	GACTTTCCCTTATCCCATCCAC	CCGAGGTTGTTGGTAAAGGTGAT
TYR	ACACACTGGAAGTATTTTTGAACA	TAGGTGCATTGGCTTCTGGG
TRP-1	TTCGTTGGAGCTGTGATTGTTG	AGGAATAATGTTGAAAGGTGGGG
TRP-2	CAGAAATAATGAGAAACTGCCAACC	TCCGTCTGCTTTATCAAACCCT
IL-6	CCCCAATTTCCAATGCTCTCC	CGCACTAGGTTTGCCGAGTA
TNF-α	CCCTCACACTCACAAACCACC	CTTTGAGATCCATGCCGTTG
GAPDH	CCTCGTCCCGTAGACAAAATG	TGAGGTCAATGAAGGGGTCGT

Supplementary Figures

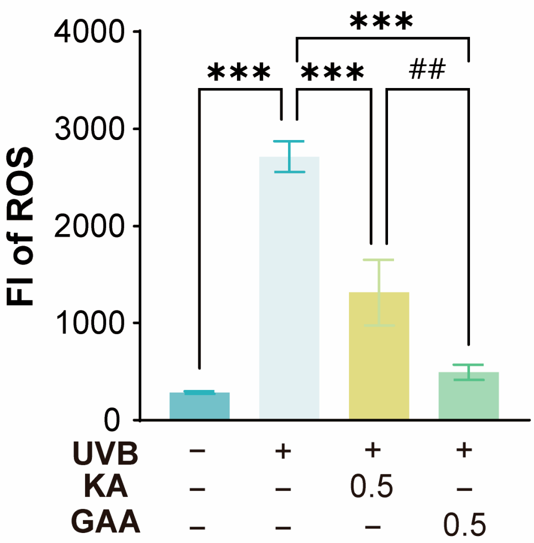

Supplementary Figure S1. Quantitative analysis of relative total ROS fluorescence intensity (n = 3).

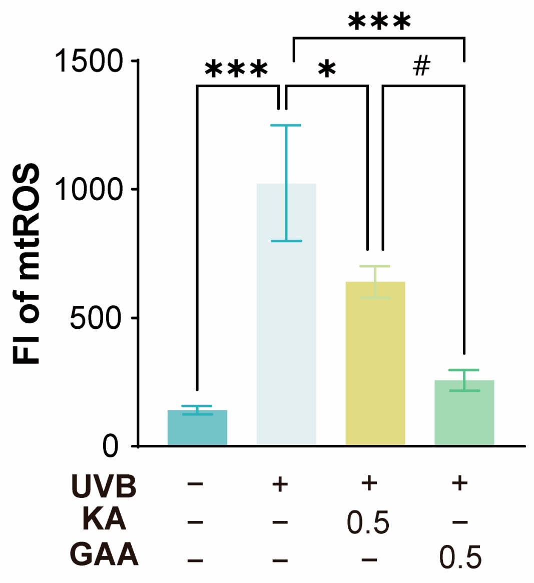

Supplementary Figure S2. Quantitative analysis of relative mtROS fluorescence intensity (n = 3).

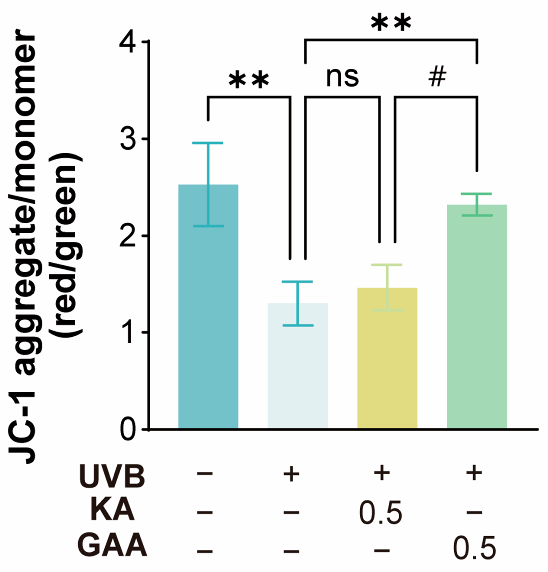

Supplementary Figure S3. Quantitative evaluation of the JC-1 aggregate/monomer fluorescence intensity ratio (n = 3).

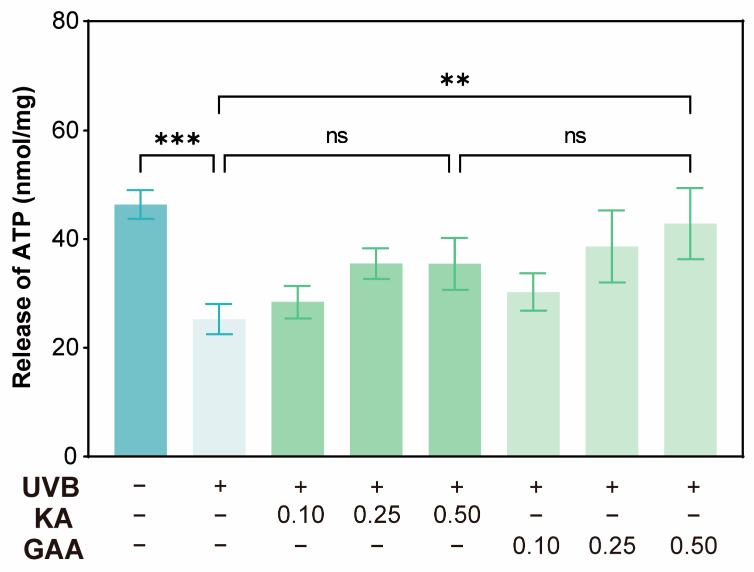

Supplementary Figure S4. Intracellular ATP content (nmol/mg protein, n = 3).

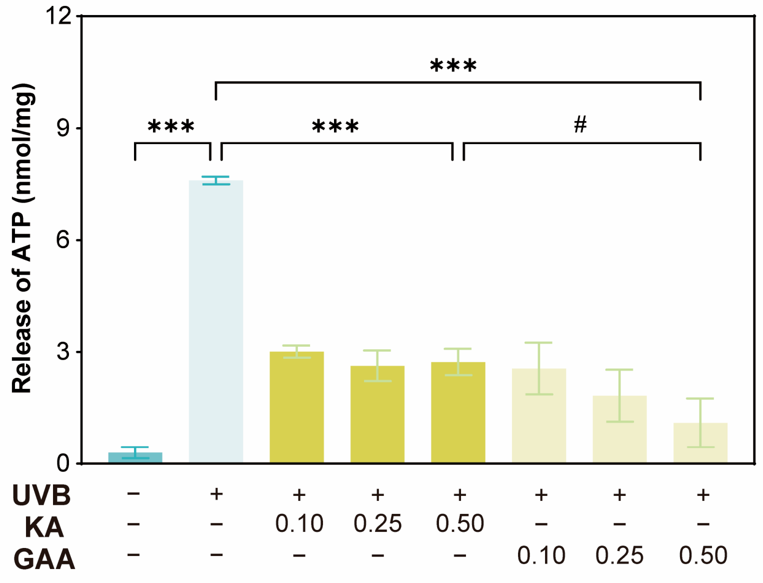

Supplementary Figure S5. Extracellular ATP release (nmol/mg protein, n = 3).

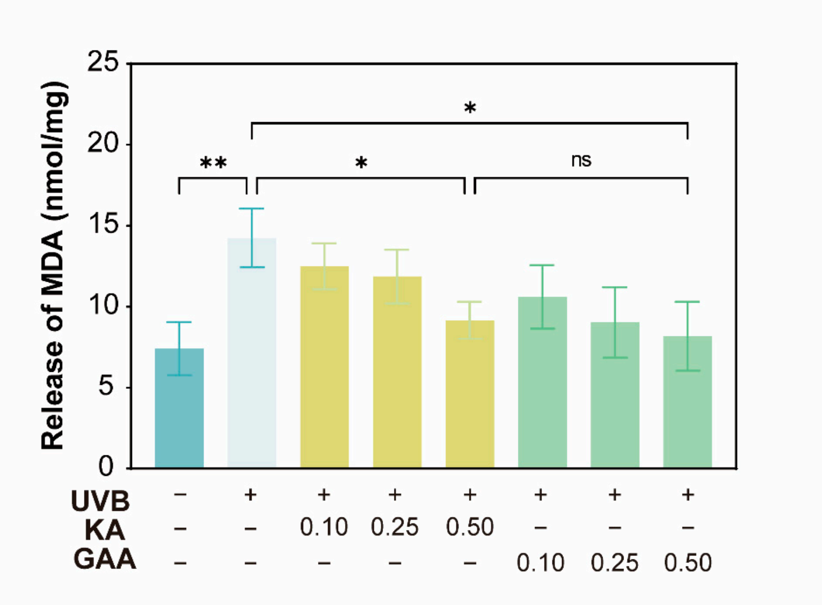

Supplementary Figure S6. Effect of KA and GAA (0.1–0.5 μmol/mL) on relative MDA levels (nmol/mg protein, n = 3).
